# Characteristics of Non-intubated Older Adult Patients With COVID-19 and Decreased Feeding and Swallowing Function and Their Association With Length of Hospital Stay

**DOI:** 10.7759/cureus.95999

**Published:** 2025-11-03

**Authors:** Shota Tanaka, Kei Goto, Kei Sakaki, Eisei Harayama, Yuya Suzuki, Chizuko Ikenaga, Kenichi Kumagae, Hisanori Oyabu, Mayu Otsuka, Kota Yamauchi

**Affiliations:** 1 Department of Rehabilitation, Steel Memorial Yawata Hospital, Kitakyushu, JPN

**Keywords:** covid-19, dysphagia, geriatrics, hospital stay, sarcopenia, swallowing

## Abstract

Background

With the increase in coronavirus disease 2019 (COVID-19) cases among older adults, the incidence of COVID-19-related dysphagia is presumed to be rising. However, reports focusing on dysphagia in non-intubated older adults with COVID-19 are rare. This study aimed to analyze the characteristics of non-intubated older adults with COVID-19 who experienced a decline in feeding and swallowing function, as assessed by the Functional Oral Intake Scale (FOIS), and examine the impact of post-COVID-19 swallowing dysfunction on the length of hospital stay.

Methodology

This retrospective study included 95 non-intubated older adults who were hospitalized with COVID-19 between May 2020 and May 2022. Patients were categorized into two groups based on the presence or absence of decreased feeding and swallowing function after the termination of isolation, as evaluated by FOIS. Intergroup comparisons were performed, and multiple regression analysis was conducted to investigate the association between impaired swallowing function at the end of isolation and the length of hospital stay.

Results

Swallowing function was impaired in 20 (21.0%) patients at the end of the isolation period. Patients with decreased feeding and swallowing function were more likely to be older, institutionalized, have higher care dependency levels, suffer from malnutrition, and have a history of dementia or neurological disease. Multiple regression analysis revealed that post-isolation swallowing dysfunction was independently associated with a longer hospital stay.

Conclusions

Older adults requiring nursing care are at a higher risk of experiencing a decline in feeding and swallowing function following a COVID-19 diagnosis, which may contribute to prolonged hospitalization.

## Introduction

Severe acute respiratory syndrome coronavirus 2 (SARS-CoV-2) causes coronavirus disease 2019 (COVID-19), which has led to a pandemic [1]. COVID-19 presents with a wide range of symptoms, including fever, dry cough, fatigue, myalgia and arthralgia, chills, headache, sore throat, and dyspnea [2]. Dysphagia is also commonly observed in patients with COVID-19 [3], and multiple factors contribute to its onset. Dysphagia is associated with the severity of COVID-19 [4,5], occurring in a large proportion of patients with severe presentation [4,6]. Acute respiratory distress syndrome, frequently observed in severe COVID-19 cases, is a major cause of endotracheal intubation in intensive care unit (ICU) patients [7,8]. Moreover, endotracheal intubation, mechanical ventilation, and tracheostomy are independent risk factors for dysphagia in ICU patients [3]. Additionally, COVID-19-related dysphagia may result from SARS-CoV-2-induced impairment of the glossopharyngeal and vagus nerves [9]. Loss of the pharyngeal reflex, pharyngolaryngeal sensory deficits, and pharyngeal contraction dysfunction are frequently observed in COVID-19-related dysphagia [9]. However, it remains unclear whether COVID-19-related dysphagia is caused by endotracheal intubation, viral infection itself, or both.

Reports on dysphagia in patients with severe COVID-19 are available, but there is a lack of studies on dysphagia in non-intubated patients with mild-to-moderate COVID-19. In clinical practice, some patients experience dysphagia despite not having severe COVID-19, highlighting the need to consider the risk of dysphagia in non-intubated patients with COVID-19 [1].

The Omicron mutant strains, circulating in recent years, have been reported to be highly infectious and transmissible [10]. The high infectivity of Omicron has led to cluster outbreaks in healthcare and nursing homes, with a rising number of infections among older adults. Swallowing function generally declines with age, and aging is a significant risk factor for dysphagia [11]. Given the increase in infections among older adults during the Omicron wave, the risk of developing COVID-19-related dysphagia may be higher. However, to our knowledge, there are no reports focusing specifically on COVID-19-related dysphagia in non-intubated older adults. Furthermore, dysphagia in patients with severe COVID-19 has been associated with prolonged hospital stays [12]. Similarly, even in patients with non-severe COVID-19, dysphagia may lead to a longer hospital stay.

Based on this evidence, we hypothesized that older adults have a higher risk of developing COVID-19-related dysphagia, which may contribute to prolonged hospitalization. This study aimed to analyze the characteristics of non-intubated older adults with COVID-19 with decreased feeding and swallowing function, as assessed by the Functional Oral Intake Scale (FOIS), and to investigate the impact of COVID-19-related decline in feeding and swallowing function on the length of hospital stay.

## Materials and methods

Study design

This study was a single-center, retrospective, cohort study.

Participants and setting

**Figure 1:**
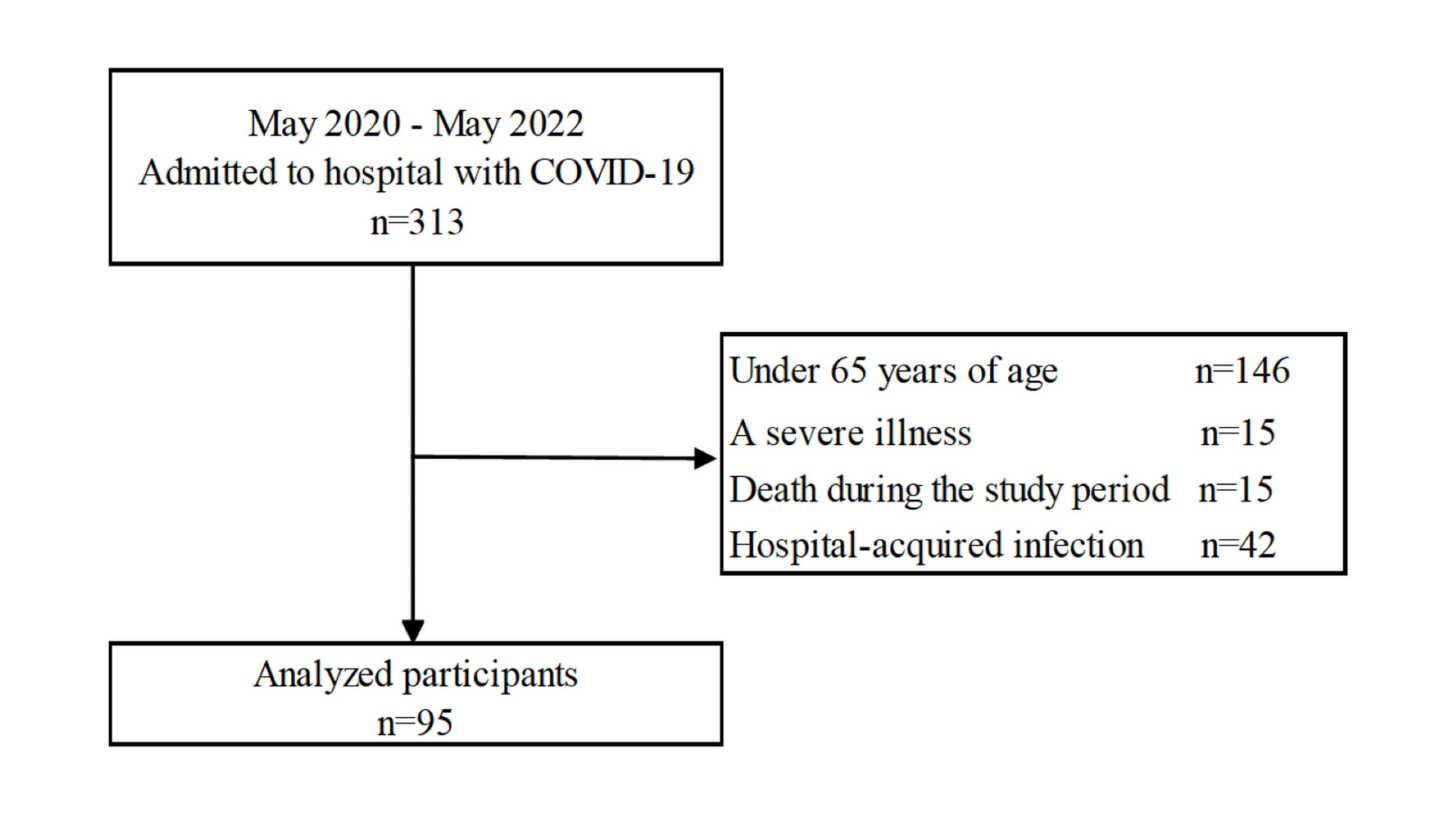
Flowchart of participant enrollment.

A total of 313 consecutive patients admitted to Steel Memorial Yawata Hospital for COVID-19 treatment between May 2020 and May 2022 were enrolled. Subsequently, 218 patients were excluded based on the following criteria: (1) age <65 years, (2) severe COVID-19, (3) death during hospitalization, and (4) COVID-19 onset during hospitalization for other conditions. These exclusions were chosen to focus on an older, non-severe, community-onset cohort and to reduce confounding from ICU/intubation-related dysphagia and informative censoring due to in-hospital mortality. Consequently, 95 patients were included in the study (Figure 1).

This study was conducted in accordance with the tenets of the Declaration of Helsinki. The patient names were coded to ensure that they could not be identified. The need for informed consent was waived due to the retrospective nature of this study. This study was approved by the Ethics Committee of Steel Memorial Yawata Hospital (approval number: 23-54; approval date: August 28, 2023).

Data collection

Data on age, sex, body mass index (BMI), duration of isolation, length of hospital stay, pre-admission residence, care category, COVID-19 severity, presence or absence of oxygen equipment, comorbidities, physiological tests, physiotherapy, and speech therapist interventions during the isolation period were retrospectively obtained from medical documents.

COVID-19 Severity Classification

COVID-19 severity was classified by the attending physicians according to the national guideline in effect during the study period. We considered severe disease as the presence of any of the following: requirement for ICU-level care, invasive or non-invasive mechanical ventilation, or rapidly progressive respiratory failure judged to need advanced respiratory support. Non-severe disease included patients managed without the above supports (i.e., room air or low-flow oxygen).

Nutritional Status

Nutritional status was assessed using the Controlling Nutritional Status (CONUT) indices. The CONUT score is calculated by scoring albumin and total cholesterol level, and total lymphocyte count, and summing the three scores [13]. The CONUT score ranges from 0 to 12, with higher values indicating worse nutritional status [13].

Long-Term Care Insurance

The need for care pre-COVID-19 was assessed using the care level. Long-term care insurance was introduced in Japan in April 2000, making all Japanese individuals aged ≥65 years eligible for benefits based on physical or mental disability. Trained local government officials assessed care needs through household visits using questionnaires on physical and mental status (73 items) and medical treatment use (12 items). The survey results were entered into computer software, standardized scores were calculated for seven items related to physical and mental condition, and the time required for nine care items (grooming/bathing, eating, toileting, transfers, meals, assistance with activities of daily living, behavioral problems, rehabilitation, medical services) was estimated. The estimated level of care was then calculated from the total care time [14]. Based on the results, patients were classified into seven levels: those who did not require constant care but needed assistance with activities of daily living (support required 1 or 2) and those who were bedridden or had dementia and required constant care (care levels 1-5).

Feeding and Swallowing Functions

Feeding and swallowing functions were assessed using the FOIS [15]. FOIS is a validated seven-point scale assessing nutritional intake, including enteral feeding, regular and modified oral intake; the scale ranges from 1 (nothing by mouth) to 7 (total oral diet with no restrictions) [15]. FOIS was assessed at the following three predefined time points: on admission, at release from isolation, and at hospital discharge. Assessments were performed by an experienced speech-language pathologist using a standardized protocol. The pre-COVID-19 FOIS score was evaluated by an experienced speech-language pathologist based on medical records and information from caregivers and family members. Disagreements were adjudicated by a senior speech-language pathologist. Formal inter-rater reliability was not measured due to infection-control constraints. A decrease in the FOIS score after release from isolation, compared with the pre-COVID-19 FOIS score, was defined as decreased feeding and swallowing function [16,17].

Statistical Analysis

Parametric data are presented as mean ± standard deviation; non-parametric data are presented as median (25th-75th percentile or interquartile range); and categorical data are presented as percentages (%). The Shapiro-Wilk test was used to assess the normality of each variable distribution. Participants whose FOIS scores decreased from before COVID-19 onset to after isolation release were classified as having decreased feeding and swallowing function, whereas those whose scores did not decrease were classified as not having decreased feeding and swallowing function. Unpaired t-, chi-squared, and Mann-Whitney U-tests were used to compare the survey items between the two groups. Single and multiple regression analyses were employed to determine whether decreased feeding and swallowing function at the time of COVID-19 isolation release was associated with the length of hospital stay. The length of hospital stay was used as the dependent variable. Decreased feeding and swallowing function at isolation release and age, BMI, CONUT score, and pre-COVID-19 FOIS score, which influence the length of hospital stay, were used in the model as adjustment variables. SPSS version 23 (IBM Corp., Armonk, NY, USA) was used for statistical analysis, with a statistical significance at p-values <0.05.

## Results

Baseline characteristics

The baseline characteristics of the enrolled participants are shown in Table 1.

**Table 1:** Participant characteristics. Continuous variables are presented as mean ± SD. Categorical variables are presented as numbers (%). Continuous variables are presented as medians. BMI: body mass index; CONUT: Controlling Nutrition Status; NHF: nasal high flow, CRP: C-reactive protein; LDH: lactate dehydrogenase; WBC: white blood cell; FOIS: Functional Oral Intake Scale; SD: standard deviation

Items	Total (n = 95)	Decreased feeding and swallowing function (n = 20)	Non-decreased feeding and swallowing function (n = 75)	P-value
Age (years)	78.0 (70.5–86)	86.5 (82.5–97.3)	76.7 (75.0–82.0)	<0.01
Males, n (%)	51 (54%)	12 (60%)	39 (52%)	0.52
BMI (kg/m²)	21.9 ± 4.3	17.6 ± 2.7	23.1 ± 4.0	<0.01
CONUT score (points)	6 (4–7)	8 (6–10)	5 (4–6)	<0.01
Length of isolation (day)	11 (9–15)	11 (9–16)	11 (10–15)	0.77
Length of hospital stay (day)	14 (10–21)	28 (19–37)	12 (10–16)	<0.01
Residence before admission, n (%)				
Home	74 (78%)	6 (30%)	68 (91%)	<0.01
Nursing facility	21 (22%)	14 (70%)	7 (9%)	
Care category, n (%)				
None	59 (62%)	5 (25%)	54 (72%)	<0.01
Support required 1	1 (1%)	0 (0%)	1 (1%)	
Support required 2	4 (4%)	1 (5%)	3 (4%)	
Care level 1	11 (12%)	2 (10%)	9 (12%)	
Care level 2	9 (9%)	3 (15%)	6 (8%)	
Care level 3	3 (3%)	2 (10%)	1 (1%)	
Care level 4	5 (5%)	4 (20%)	1 (1%)	
Care level 5	3 (3%)	3 (15%)	0 (0%)	
Severity, n (%)				
Mild	27 (28%)	6 (30%)	21 (28%)	0.56
Moderate disease I	33 (35%)	5 (25%)	28 (37%)	
Moderate disease II	35 (37%)	9 (45%)	26 (35%)	
Oxygen devices, n (%)				
NHF	2 (2%)	1 (5%)	1 (1%)	0.38
Nasal cannula	49 (52%)	12 (60%)	37 (49%)	0.40
Coexisting disease, n (%)				
Respiratory illness	10 (11%)	3 (15%)	7 (9%)	0.35
Cerebral neurological disease	24 (25%)	11 (55%)	13 (18%)	<0.01
Dementia	30 (32%)	17 (85%)	13 (17%)	<0.01
Psychotropic drug	8 (8%)	2 (10%)	6 (8%)	0.54
Physiological tests				
CRP (mg/dL)	4.1 (1.9–8.1)	4.5 (2.4–13.0)	4.0 (1.6–7.2)	0.20
LDH (U/L)	249 (207–317)	248 (192–305)	256 (210–323)	0.12
WBC (×10³/μL)	5.29 (4.00–6.80)	6.60 (4.89–9.50)	5.01 (4.00–6.42)	<0.05
Rehabilitation, n (%)				
Physical therapy	44 (46%)	15 (75%)	29 (39%)	<0.01
Feeding therapy	1 (1%)	1 (0.5%)	0 (0%)	0.21
FOIS score (points)				
Before admission	7 (6–7)	6 (5–7)	7 (6–7)	<0.01
Removal of isolation	7 (6–7)	1 (1–5)	7 (6–7)	<0.01
Time of discharge	7 (6–7)	5 (1.75–5)	7 (6–7)	<0.01

The participants’ median age was 78.0 (70.5-86.0) years, with 51 (54%) being male. The BMI was 21.9 ± 4.3 kg/m², and the pre-COVID-19 FOIS score was 7 (6-7). The isolation duration was 11 (9-15) days, and the length of hospital stay was 14 (10-21) days. Of the 95 patients, 20 (21.0%) had decreased feeding and swallowing function at isolation release. Among the nine patients with normal function (FOIS score, 7) before COVID-19, four transitioned to non-oral feeding (FOIS score, 1), and five were still able to orally ingest food, but had transitioned to decreased feeding and swallowing (FOIS score, 5-6). Among the 11 patients who were able to eat orally but had decreased feeding and swallowing (FOIS score, 5-6) before COVID-19, eight had transitioned to non-oral feeding (FOIS score, 1) (Table 2).

**Table 2:** Change of feeding and swallowing function before and after COVID-19 diagnosis. FOIS: Functional Oral Intake Scale

		Post-COVID-19 FOIS score			
		1	5	6	Total
Pre-COVID-19 FOIS score	5	6	0	0	6
	6	2	3	0	5
	7	4	1	4	9
Total		12	4	4	20

Characteristics of patients with decreased feeding and swallowing function at isolation

The group with decreased feeding and swallowing function was significantly older and had a lower BMI, lower pre-COVID-19 FOIS score, and a higher CONUT score compared to the non-decreased feeding and swallowing function group (all p < 0.01). The patients with decreased feeding and swallowing function were more likely to have been institutionalized before admission and required higher care levels (all p < 0.01), compared with those without decreased feeding and swallowing function. The group with decreased feeding and swallowing function also had a higher prevalence of pre-existing neurological disorders and dementia, compared with the group without decreased feeding and swallowing function (all p < 0.01). There were no significant differences in COVID-19 severity (p = 0.56) or oxygen equipment use (nasal high flow; p = 0.38, nasal cannula; p = 0.40). The FOIS scores were significantly lower in the decreased feeding and swallowing function group at discharge (p < 0.01) (Table 1).

Relationship between decreased feeding and swallowing function and length of hospital stay

There was no significant difference in isolation duration between the groups (p = 0.77); however, the length of hospital stay was significantly longer in the decreased feeding and swallowing function group than in the group without decreased feeding and swallowing function (p < 0.01). Single regression analysis showed an association between feeding and swallowing function at isolation release and the length of hospital stay (β = 0.590, 95% confidence interval (CI) = 11.949-21.364, p < 0.01). After adjusting for age, BMI, CONUT score, and pre-COVID-19 FOIS score, decreased feeding and swallowing function at isolation release remained an independent factor associated with the length of hospital stay (β = 0.505, 95% CI = 8.293-20.260, p < 0.01) (Table 3).

**Table 3:** Multiple regression analysis of length of hospital stay. BMI: body mass index; CI: confidence interval; CONUT: Controlling Nutrition Status; FOIS: Functional Oral Intake Scale

	β	95% CI	P-value
Decreased feeding and swallowing function	0.505	8.293 to 20.260	<0.001
Age	0.188	-0.044 to 0.482	0.102
BMI	0.128	-0.191 to 0.875	0.205
CONUT score	0.173	-0.113 to 1.828	0.083
Pre-COVID-19 FOIS score	0.056	-1.698 to 2.891	0.607

## Discussion

In this study, 20 (21.0%) non-intubated patients with COVID-19 exhibited decreased feeding and swallowing function at the time of isolation release. The results showed that patients with decreased feeding and swallowing function at discharge from isolation were characterized by advanced age, institutionalization, high care needs, poor nutritional status, and a history of dementia or neurological disease. Additionally, multiple regression analysis revealed that decreased feeding and swallowing function at the time of isolation release influences the length of hospital stay. This finding suggests that, even after adjusting for factors related to the length of stay, decreased feeding and swallowing function at isolation release remains an independent factor associated with the length of hospitalization.

COVID-19 presents with a variety of clinical symptoms, and dysphagia is one of the common symptoms observed in patients with COVID-19. Older adults with COVID-19 may experience severe outcomes, and a relatively high proportion require hospitalization and ICU management [3]. Patients with severe COVID-19 who require ICU admission may need some form of respiratory support, including endotracheal intubation and mechanical ventilation. In ICU patients, endotracheal intubation, mechanical ventilation, and tracheostomy are all independent risk factors for dysphagia [3]. Furthermore, patients with severe COVID-19 are more likely to develop dysphagia due to conditions such as cerebrovascular events, encephalitis, encephalopathy, peripheral neuropathy, and myositis [9].

Dysphagia in non-intubated patients with COVID-19 has also been reported, with 20% of such patients (median age = 51.66 years) developing dysphagia during hospitalization [18]. This suggests an association with changes in pulmonary respiratory function and direct neurological impairment caused by the virus [18], indicating that dysphagia can occur even in non-severe cases. In this study, although the decline in feeding and swallowing function was assessed using the FOIS, its prevalence was 21%, which is consistent with previous reports. These findings suggest that similar factors may be associated with dysphagia in non-intubated patients with COVID-19. Research investigating COVID-19-related dysphagia has mainly focused on severe intubated patients, but non-intubated patients may also develop swallowing dysfunction due to several factors. Even in the absence of endotracheal intubation or neurological disorders, sarcopenic dysphagia can occur in older adults [1]. Sarcopenic dysphagia arises from the weakness of swallowing-related muscles caused by sarcopenia. There is an association between care dependency and sarcopenia, with the risk of requiring care being higher in those with sarcopenia [19]. Additionally, the prevalence of sarcopenia increases with age, particularly in both men and women over the age of 75 years [20]. While this study is a retrospective cohort study focusing on patients with COVID-19 and does not include an assessment of sarcopenia, a higher proportion of care-dependent individuals and older adults was observed in the decreased feeding and swallowing function group. Moreover, the decreased feeding and swallowing function group had significantly higher rates of dementia [21] and neurological disorders [22], both of which are strongly associated with sarcopenia. These findings suggest that older adults who were already in a care-dependent state and residing in long-term care facilities may have developed secondary sarcopenia and sarcopenic dysphagia due to the inflammation from COVID-19, nutritional deficiencies during hospitalization, and reduced physical activity resulting from several days of isolation. In older adults with COVID-19, sarcopenia, along with the effects of the virus, may contribute to the development of dysphagia.

Malnutrition in older adults is associated with dysphagia [23] and affects outcomes across various diseases [24]. COVID-19-related dysphagia increases the risk of pulmonary complications, such as aspiration pneumonia, potentially leading to malnutrition and worsening outcomes [9], which supports the findings of this study.

In this study, no difference was observed in the number of days to release from isolation; however, the length of hospital stay was 2.3 times longer in patients with decreased feeding and swallowing function than in those without decreased feeding and swallowing function. Multiple regression analysis also revealed that decreased feeding and swallowing function at the time of isolation release was associated with the length of hospital stay. The results suggest that decreased feeding and swallowing function at isolation release independently influences the length of hospital stay, even after adjusting for related factors. During the pandemic peak, hospitals were overwhelmed with critically ill patients requiring admission, leading to shortages of staff, beds, and supplies, and increasing the number of patients awaiting admission despite maximum hospital capacity [25]. Additionally, reducing the length of hospital stay is crucial, especially for older adults, as prolonged hospitalization for acute illnesses contributes to declines in physical function and activities of daily living [26,27].

Difficulties in rehabilitation and nutritional interventions due to infection control measures further decrease physical function in patients with COVID-19, resulting in disuse muscle atrophy [9]. This can cause dysphagia and prolong the length of hospital stay. However, for patients testing positive for COVID-19, non-contact interventions, such as dietary modifications and telemedicine, are typically prioritized over direct active exercises until the infection resolves [28]. At our hospital, due to infection control measures, rehabilitation interventions during the isolation period were largely limited. It is crucial to identify patients at risk for dysphagia and to minimize this risk. The results of this study suggest the importance of early rehabilitation interventions and nutritional therapy for older adults with COVID-19. Moreover, this study may provide valuable insights for future management of similar novel infectious diseases requiring isolation measures.

This study has several limitations. First, this was a single-center, retrospective study, which hindered the generalization of results or removal of potential confounders; therefore, no firm conclusions could be drawn. Second, the FOIS was used to assess feeding and swallowing function. This instrument evaluates the level of oral intake rather than dysphagia per se; owing to infection-control requirements and limited resources during the pandemic, the gold-standard videofluoroscopic examination of swallowing (VF) [29] could not be performed. Formal assessment of inter-rater reliability for FOIS was not conducted; although evaluations followed a standardized protocol by experienced speech-language pathologists, infection-control constraints may have introduced measurement bias. In addition, the pre-COVID-19 FOIS was ascertained retrospectively from medical records and caregiver/family reports, which may be subject to misclassification. These limitations may have influenced the results. Third, sarcopenia was not assessed in this study due to reasons similar to those that precluded the use of VF. Lastly, the COVID-19 pandemic itself may have affected the length of hospital stay. Factors that were not adjusted for in this study, such as clustering of discharge sites and COVID-19 diagnosis among caregivers, may have affected the length of hospital stay.

## Conclusions

Among non-intubated patients with COVID-19, 21.0% developed decreased feeding and swallowing function at the time of isolation release. The characteristics of these patients included older age, residence in long-term care facilities, high care dependency, malnutrition, and a history of dementia and neurological disorders. Additionally, decreased feeding and swallowing function at the time of isolation release may be associated with prolonged hospital stays.
